# The Doctor and His Patient

**Published:** 1920-06-12

**Authors:** 


### THE PUBLIC WELL-BEING.

### The Doctor and his Patient.

### The Advisory Relationship.

A STORY was told a short time ago, in one of the hospital magazines, of the day's work of a medical officer in Nigeria, and it included the tale of a visit from a native woman with her baby. The child was not ill, but the mother had heard of the white doctor's medicine, so she thought she would like to have some to prevent the child from getting ill in the future. This is a crude illustration of an important point, and it shows a certain degree of native wisdom which our higher civilisation might do well to follow in principle. How many parents take their children to medical advisers when the children are well? People who attend the dentist for a regular overhaul, and doubtless send their children for the same purpose, rarely follow this practice with the doctor. The State has realised the value of it, and in the medical inspection of children, from the nursery-school period right on to the end of school life, provision for the regular examination of school children is to some extent provided for. There is far more understanding of the signs of ill-health than there used to be, and the intelligent person—if for himself he does not at once seek some remedy—has sufficient solicitude for his children or others in his circle to see that they do not go without medical attention. But, even then, there are masses of people who are very casual, and have ready explanations for ailments which may be the beginnings of more serious things. No one would advise, without any sense of proportion, a perpetual rush to the general practitioner, but it would be a great gain if people made a point of having regular times—say, once a year at the most—for a general overhaul for themselves and their young people; it is for the young that it would be so valuable. As for the adults, most of them would never be persuaded, when in apparent good health, to get medical advice, for so many entirely ignore all physical warnings. The record of Army examinations showed the great number of men suffering from serious diseases, but absolutely ignorant of the fact until compelled to undergo examination. The extent of such cases of serious disease would have been considerably lessened if resort to medical advice had been more general.

#### AN AMERICAN SUGGESTION.

There is another very interesting side of this matter which is presented in an article in the *Long Island Medical Journal*, on what a child should demand of his doctor. This article, written by a New York doctor, asks what medical practitioner can honestly say that he has no child in his practice with frankly undesirable tonsils or obstructive adenoid vegetations—or with a marked astigmatism uncorrected by glasses, or with some other gross error plainly discernible for him who runs to read—which he has never suggested be corrected because the parent has never directly asked his counsel in the matter. How many ever urge protective work for the children, or need more than the fingers of one hand on which to count the number of families, or even of individuals, that come to them in what they believe to be good health, for a periodical overhaul? The article voices a plea for four things. First, a complete, detailed, recorded history, and physical and mental examination, of every child within the reach of every practitioner who has to do with children, either in private practice or in dispensary work. Secondly, a clearly written out, simply worded summary of the child's condition, with definite, comprehensive recommendations as to his future management. Thirdly, a campaign of propaganda of the very best sort—the person-to-person, doctor-to-mother kind. And, fourthly, a periodical re-examination and checking-up of results, to show the easily demonstrable improvement that such work is bound to produce, and to encourage both doctor and parent to continue in the work of betterment.

The writer believes that most doctors have at some time or other been genuinely embarrassed, and actually at a loss as to just how to proceed, when a father or mother has brought a child with the request for a complete examination, to be repeated from time to time—surely not an unreasonable request, and quite as surely indicative of a spirit that ought to be encouraged. And yet has not every medical man failed to give such a parent the service he desired, and actually discouraged him from returning at subsequent regular intervals, by the plainly evident uncertainty and lack of method or system with which this uncongenial, because unusual, task is approached?

#### REGULAR OVERHAUL AND RECORDS.

"How easy it would have been" (says the article) "to have fostered this advisory relationship, with all of the good that it would have meant to the child, had we but had a definite line of procedure to follow, instead of our usual rather aimless pulling down of eyelids for anæmia and cursory chest examination, with perhaps a request for a specimen of urine, that as a rule ends the seance. We are quick to respond to the call for the treatment of disease that produces symptoms, but as a rule are quite unprepared to look for health, and evaluate it as improvable or not. Some such definite procedure, completed by some form of report by which doctor and parent would know just where the child stood, and whether he was improving, stationary, or retrogressing, would have cemented this relationship, and shown both doctor and parent what to stress and what to be satisfied with in their joint efforts to improve the condition of the child. Much more, in the way of regularly and periodically ascertaining and showing the parent progress in height, chest expansion, general nutrition, orthopædic correction, and a dozen other vital particulars, is essential, if we are

**THE PUBLIC WELL-BEING—(*continued*)**

to gain and hold the individual child long enough to give him the service that he is entitled to. All of this can be put into readily assimilable form for the parent, in such a way as to make intensely interesting records for him, as well as most valuable data for the observing and directing physician."

This is all most suggestive, and it is, in a sense, complementary to a point put forward in these columns some time ago in relation to the question of health statistics. It was then asked whether it would not be possible for the general practitioner to keep records and make returns of the central health facts observed in his practice. The article in the American journal treats the same point from the angle of the individual patient, and if his ideas were carried out in general the national statistical record would follow very simply. But the proposals are difficult, involving, as they do, such a considerable addition to the work of the doctor. They are, however, worth consideration, as are all suggestions whose object is to raise the general standard of health, and they lend solid support to the idea of a regular overhaul.

